# Micronucleus abnormalities and sex differences in medical staff exposed to occupational ionizing radiation: a retrospective cohort study

**DOI:** 10.3389/fpubh.2025.1730667

**Published:** 2025-12-12

**Authors:** Gaiyan Du, Yuanling Wu, Huiqin Zhang, Zhenxin Guo, Jingyi Wu, Bozheng Zhang, Junyan Zhang, Jing Wang, Junkang Zhao, Ruijuan Zhang, Ruonan Duan, Fang Gao

**Affiliations:** 1 Shanxi Bethune Hospital, Shanxi Academy of Medical Sciences, Tongji Shanxi Hospital, Third Hospital of Shanxi Medical University, Taiyuan, China; 2 Emory University Emory College of Arts and Sciences, Atlanta, GA, United States

**Keywords:** occupational radiation, micronucleus assay, sex differences, cytogenetic damage, healthcare workers, retrospective cohort study

## Abstract

**Background:**

Long-term occupational exposure to low-dose ionizing radiation increases the risk of genetic damage among medical staff. Micronucleus (MN) frequency is a sensitive biomarker of chromosomal damage and genomic instability, but the influence of sex on MN responses to chronic radiation exposure remains insufficiently understood.

**Objective:**

This study aimed to assess sex – related differences between male and female healthcare workers in MN frequency abnormalities among those chronically exposed to ionizing radiation and to explore potential biological and occupational determinants underlying these differences.

**Methods:**

This retrospective cohort study included 102 medical staff (65 males, 37 females) from Shanxi Bethune Hospital, who had documented occupational radiation exposure for at least 10 years (2012–2024). According to the Chinese national standard GBZ/T 328–2023, a micronucleus frequency of ≥6‰, that is, ≥6 micronuclei per 1,000 binucleated lymphocytes, was classified as abnormal, indicating elevated chromosomal damage. Poisson regression analysis was performed to examine predictors of MN abnormalities, adjusting for age, cumulative radiation dose, and occupational category.

**Results:**

Females had a significantly higher rate of MN frequency abnormalities than males (11.0 vs. 5.6 per 100 person-years, *p* = 0.008). In the multivariate Poisson regression analysis, female sex remained significantly associated with MN abnormalities (adjusted Coef. = 0.636, 95% CI: 0.176–1.096, *p* = 0.007). However, interpreting female sex as a strictly biological risk factor is limited by its correlation with occupational roles, most notably, the higher proportion of nurses among females. Furthermore, the absence of smoking and alcohol use data in the female subgroup complicates the assessment of behavioral confounding. In contrast, cumulative radiation dose during the study period was not significantly associated with MN abnormalities in the multivariate model (*p* > 0.05).

**Conclusion:**

After adjustment for measurable confounders, female healthcare workers exhibited significantly higher levels of cytogenetic damage. This association may reflect a combination of biological susceptibility, unmeasured occupational exposures, and behavioral factors—rather than cumulative physical radiation dose alone. These findings support including sex as a biological variable in occupational radiation safety protocols to improve risk stratification. They also highlight the need for future research to disentangle biological sex effects from correlated occupational and lifestyle determinants.

## Graphical abstract

**Figure:**
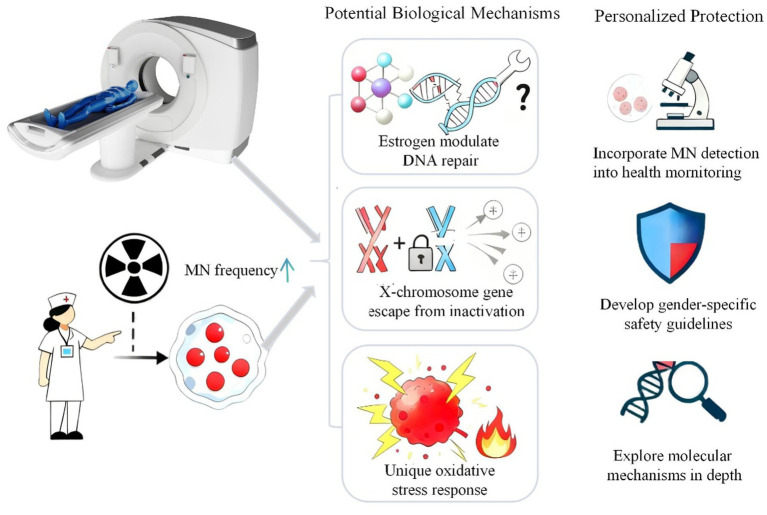

## Introduction

Chronic occupational exposure to low-dose ionizing radiation is a significant concern for healthcare workers in fields such as radiology, interventional cardiology, and nuclear medicine. These workers are routinely exposed to scattered radiation during diagnostic and therapeutic procedures. This exposure increases the risk of cumulative genetic damage over a professional lifetime, even when personal dosimetry remains within annual regulatory limits (1). Therefore, sensitive biological indicators are essential for detecting early cytogenetic changes and assess individual susceptibility beyond what physical dose measurements can capture.

The cytokinesis-block micronucleus (CBMN) assay is a robust tool for this purpose (2). It quantifies MN that form from acentric chromosome fragments or lagging whole chromosomes during cell division. This provides a direct measure of chromosomal damage and genomic instability, where elevated MN frequency not only indicates recent genotoxic exposure but also predicts an increased risk for cancer and other complex diseases in epidemiological studies.

Owing to its sensitivity and reliability, the CBMN assay has become a core tool for biological dosimetry and for monitoring cytogenetic damage in populations occupationally exposed to ionizing radiation (3). Studies among medical radiation workers, particularly in interventional radiology and nuclear medicine, frequently report elevated MN frequencies, reinforcing the genotoxic risk of chronic low-dose exposure (4, 5). However, a critical limitation persists in this body of literature: the majority of these studies have either predominantly enrolled male participants or failed to conduct sex-stratified analyses (6). This androcentric bias means that the potential for sex-specific differences in radiosensitivity has been largely overlooked, impairing a mechanistic understanding and the development of equitable (EDI) health policies. While inter-individual variability is acknowledged, the specific contribution of biological sex remains a glaring gap.

Biological and epidemiological evidence supports the rationale for examining sex differences. For example, hormonal influences such as estrogen signaling modulate DNA repair proteins including BRCA1, RAD51, and XRCC1 (7–9), indicating that DNA repair capacity differs between sexes. Genetic factors, such as the dosage of X-linked genes and incomplete X-chromosome inactivation may further influence DNA damage response and antioxidant defense mechanisms in females. Epidemiological data also show higher rates of radiation-induced toxicities among female radiotherapy patients (10), and greater cytogenetic damage among female workers exposed to occupational radiation (11, 12), suggesting heightened female susceptibility.

Despite these indications, few studies have directly compared MN frequencies between male and female healthcare workers exposed to chronic low-dose ionizing radiation. Therefore, this study aims to systematically evaluate sex-related differences in MN abnormalities among radiation-exposed healthcare workers and tests the hypothesis that females exhibit higher MN frequencies independent of cumulative radiation dose. The ultimate objective is to generate evidence that supports the development of personalized and equitable radiation safety protocols that account for sex-specific biological differences.

## Materials and methods

### Study design and population

This retrospective cohort study analyzed occupational health examination records of medical staff exposed to ionizing radiation at Shanxi Bethune Hospital. The records spanned from February 1, 2012, to April 30, 2024.

#### Sample size consideration

An *a priori* power analysis was conducted to ensure sufficient statistical power for detecting sex-based differences in MN abnormality rates. Based on prior occupational cytogenetic studies, we assumed MN abnormality rates of 5% for males and 10% for females. Using a two-sided significance level (*α*) of 0.05 and a statistical power (1 − *β*) of 0.80, we calculated the minimum required sample size using the two-proportion *z*-test formula.


n=(Zα2+Zβ)2×[p1(1−p1)+p2(1−p2)](p1−p2)2

where Z_α_/₂ = 1.96 and Zᵦ = 0.84. The resulting minimum required sample size was 61 participants per group (total n ≈ 122).

### Participant selection and inclusion criteria

**Figure 1:**
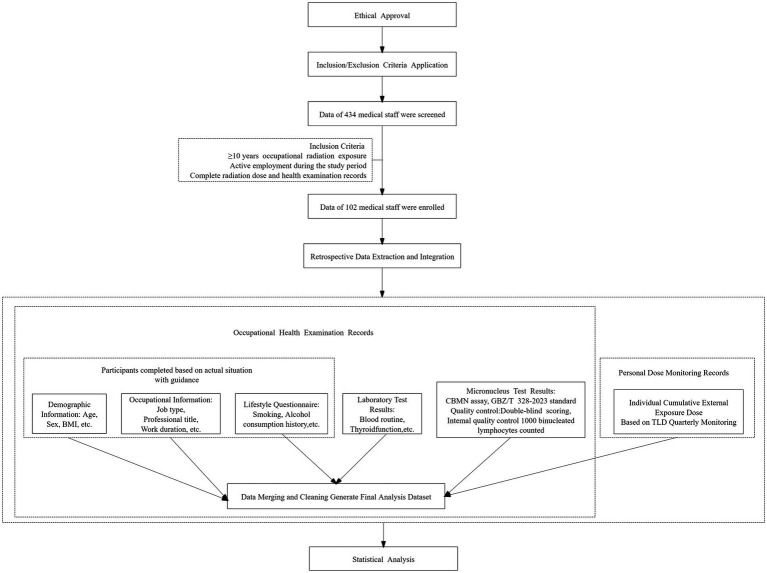
The flow diagram of participants enrollment.

From an initial screening of 434 individuals, 102 participants met all inclusion criteria. Among them, 65 were males and 37 were females. These criteria included at least 10 years of documented occupational radiation exposure, active employment during the study period (2012–2024), availability of complete radiation dose monitoring data, and availability of standardized occupational health examination records. Notably, smoking and alcohol consumption were not used as exclusion criteria, as one aim of the study was to assess the independent effect of sex while accounting for these potential behavioral confounders in the analysis (see Figure 1).

### Data collection and variable definition

Data were systematically collected from the institutional occupational health database. Collected variables included age, sex, body mass index (BMI), education level, job title, and occupational category (physician, nurse, radiologic technologist).

Additionally, participants were categorized based on their main type of radiation work, specifically reflecting their dominant source of occupational exposure. These modalities included diagnostic X-ray, computed tomography (CT); interventional procedures; nuclear medicine; and radiotherapy. This classification was used to characterize the cohort composition and assess potential exposure heterogeneity related to different radiographic equipment and procedures.

### Lifestyle factors were assessed using standardized questionnaires

During the occupational health examination, which mainly evaluated established behavioral risk factors with genotoxic effects, such as smoking and alcohol consumption. Smoking status was classified into three groups: never smokers (fewer than 100 cigarettes in a lifetime), former smokers (at least 100 cigarettes in a lifetime but none in the past 12 months), and current smokers (13, 14).

Alcohol consumption was categorized into three groups: non-drinkers, occasional drinkers, and regular drinkers. Non-drinkers reported never drinking or fewer than 10 total drinking occasions, occasional drinkers consumed alcohol non-weekly, up to 4 times per month, with less than 1 standard drink (10 g pure alcohol) per occasion; regular drinkers consumed alcohol weekly for at least 6 months or 5 or more times per month with at least 1 standard drink per occasion (15).

However, data on other exposure sources—such as specific dietary habits, hobbies involving chemical solvents or pigments, non-occupational radiation, and detailed residential history—were not systematically collected in the current occupational health database. This limitation prevented assessment of their influence in this study.

The research team conducted all health examinations and laboratory tests according to the Chinese national standard GBZ 98–2020. This standard specifies the health criteria and surveillance program for radiation workers and incorporates the fundamental principles for medical monitoring of occupational radiation exposure as recommended by international bodies such as the ICRP and IAEA.

### Radiation exposure assessment

Personal cumulative external radiation dose measurements were obtained through routine quarterly monitoring. Thermoluminescent dosimeters (TLDs, model IDB-3, and detection limit 0.01 mSv) were worn on the left chest beneath lead aprons. The procedures for personal dose monitoring and quality control complied with the Chinese national standard GBZ 207–2016. This standard is technically aligned with the relevant International Atomic Energy Agency (IAEA) guidelines and incorporates the fundamental principles of the International Commission on Radiological Protection (ICRP), specifically the ALARA (As Low As Reasonably Achievable) principle.

### Two exposure metrics were defined

Years of employment in radiation work before the first examination is defined as the time from starting radiation work to the first health examination during the study period, serving as a proxy for long-term exposure history.

Cumulative effective dose during the study period is the sum of all recorded personal dose equivalents from the first to the last examination, which is the primary quantitative exposure variable. The mean annual dose is calculated by dividing this cumulative dose by the corresponding number of years.

### Management of anomalous dose readings and exclusion of non-occupational exposures

Our institution implements a strict protocol for investigating anomalous dose readings. Specifically, this protocol helps ensure the integrity of personal dosimetry data and aims to isolate the effect of chronic occupational exposure. Any worker whose recorded dose in a monitoring period exceeds expected levels triggers a formal investigation. This investigation uses a standardized “Dose Verification Registration Form” and, if necessary, an “Over-dose Investigation Form.” These forms specifically target and document potential non-routine exposures, such as accidental opening or immersion of the dosimeter, leaving the dosimeter in the radiology suite, or wearing the dosimeter during personal medical examinations. Other examples include assisting patients during examinations, repairing source-containing devices, confusion between inside and outside lead apron dosimeters, and significant transient workload increases. Crucially, no participant included in the final study cohort had ever triggered or required such an investigation during their entire follow-up period. Therefore, this process provides a high degree of confidence that the cumulative doses analyzed in our study represent routine, occupational low-dose exposure and are not confounded by acute, accidental, or non-occupational exposure events.

### Micronucleus assay

The CBMN assay was conducted using peripheral venous blood samples in strict accordance with the Chinese national standard GBZ/T 328–2023 (16). This standard aligns with the international guideline ISO 17099: 2014 (17) and the method developed by Fenech (18).

After 44 h of culture, cytochalasin B was added to arrest cells at the binucleated stage, and culture continued until 72 h. Cells were then harvested, fixed, dropped onto slides, and Giemsa-stained. MN frequency was determined by two independent, blinded evaluators scoring 1,000 binucleated lymphocytes per individual under 400 × magnification, adhering to international scoring criteria. A MN frequency of ≥ 6‰ (≥ 6 MN per 1,000 BN cells) was defined as abnormal (16), consistent with findings from international cytogenetic studies (19, 20). These studies, including data from the Human MicroNucleus (HUMN) Project (21), have established that such an elevated MN frequency indicates increased chromosomal damage.

### Potential sources of variability

The MN frequency for each participant was measured from a single blood sample. Intra-individual biological variation (e.g., transient infections, stress, or circadian rhythm) may affect the measurement. To minimize the potential impact of diurnal variation, However, all blood samples were collected in the morning. Furthermore, we strictly followed the standardized protocol (GBZ/T 328–2023) and used blinded scoring by two independent evaluators to ensure the highest reproducibility and accuracy of the measurement.

### Statistical analysis

Statistical analyses were performed using Stata SE 13, R 3.6.1, and EmpowerStats 2.0. Continuous variables were presented as mean ± standard deviation (SD) or median (interquartile range), and categorical variables were presented as counts (percentages). Intergroup comparisons were performed using Student’s t-test, Mann–Whitney U test, χ^2^ test, or Fisher’s exact test, as appropriate.

Poisson regression was used to identify independent predictors of MN abnormalities, and the results were reported as coefficients (Coef.) with 95% confidence intervals (CI). Variables with a *p*-value < 0.10 in univariate analysis were included in the multivariate model, the multivariate model was further adjusted for age, cumulative years of occupational radiation exposure work prior to the first examination, occupational category, and cumulative radiation dose during the study period. Statistical significance was defined as a two-sided *p*-value < 0.05.

### Ethical considerations

The study was approved by the Ethical Review Committee of Shanxi Bethune Hospital (Reference Number: YXLL-2024-098) and conducted in accordance with the Declaration of Helsinki. Written informed consent was obtained from all participants.

## Results

### Population characteristics

The study cohort included 102 radiation-exposed medical workers, with a mean age of 31.66 ± 6.40 years, and the mean body mass index (BMI) of 23.15 ± 3.07 kg/m^2^. Table 1 shows significant sex-based differences in BMI and occupational distribution. Male participants had a significantly higher BMI than their female counterparts (24.25 ± 2.86 kg/m^2^ vs. 21.22 ± 2.41 kg/m^2^, *p* < 0.001). Occupational roles also differed substantially: the majority of males were licensed physicians (63.1%), while the largest proportion of females were nurses (29.7%; *p* = 0.002 for the difference in occupational distribution between sexes). Moreover, smoking and alcohol consumption were more common among male staff (both *p* < 0.001). In contrast, no significant differences were observed between sexes regarding age, education level, marital status, professional rank, years of radiation exposure before the initial examination, or the prevalence of lens opacity.

**Table 1:** Demographic characteristics of all participants at the initial examination.

Variable		All (*n* = 102)	Male (*n* = 65)	Female (*n* = 37)	Statistics	*p*-value
Age	Mean (SD)	31.66 (6.40)	31.58 (6.84)	31.78 (5.62)	*t* = −0.15	0.881
	Min-Max	22–50	22–50	23–43		
Body mass index (Kg/m^2^)	Mean (SD)	23.15(3.07)	24.25 (2.86)	21.22 (2.41)	*t* = 5.44	<0.001
Education level	Associate	11 (10.78%)	8 (12.31%)	3 (8.11%)		0.113^Δ^
	Bachelor	36 (35.29%)	18 (27.69%)	18 (48.65%)		
	Master and above	55 (53.92%)	39 (60.00%)	16 (43.24%)		
Marriage	Single	42 (41.18%)	28 (43.08%)	14 (37.84%)	*χ*^2^ = 0.27	0.605
	Married	60 (58.82%)	37 (56.92%)	23 (62.16%)		
Job rank	Senior	18 (17.65%)	12 (18.46%)	6 (16.22%)	*χ*^2^ = 2.03	0.363
	Intermediate	20 (19.61%)	10 (15.38%)	10 (27.03%)		
	Junior	64 (62.75%)	43 (66.15%)	21 (56.76%)		
Employee classification	Licensed Physicians	56 (54.90%)	41 (63.08%)	15 (40.54%)		0.002^Δ^
	Nurses	14 (13.73%)	3 (4.62%)	11 (29.73%)		
	Technologists	32 (31.37%)	21 (32.31%)	11 (29.73%)		
Years of radiation exposure before the initiate examination	Median (Q1-Q3)	1 (0–5)	1 (0–7)	0 (0–4)	*z* = 0.71	0.478
	Min-Max	0–29	0–29	0–16		
Smoke		31 (30.39%)	31 (47.69%)	0 (0.00%)		<0.001^Δ^
Number of cigarettes	Median (Q1-Q3)	0 (0–1.75)	0 (0–8)	0 (0–0)	*z* = 4.70	<0.001
	Min-Max	0–20	0–20	0–0		
Drink		34 (33.33%)	34 (52.31%)	0 (0.00%)		<0.001^Δ^
Classification of Radiographic Equipment	Diagnostic X-ray + CT Scan + Radiotherapy (ClassIII)	46 (45.09%)	30 (46.15%)	16 (43.24%)	*χ*^2^ = 0.08	0.776
	Interventional Procedures + Nuclear Medicine (ClassII)	56 (54.90%)	35 (53.85%)	21 (56.76%)		
	Diagnostic X-ray + CT	44	28	16		
	Radiotherapy	11	10	1		
	Interventional Procedures	35	23	12		
	Nuclear Medicine	12	4	8		
Hashimoto’s thyroiditis		1 (0.98%)	0 (0.00%)	1 (2.70%)		0.363^Δ^
Hypothyroidism		1 (0.98%)	0 (0.00%)	1 (2.70%)		0.363^Δ^
Lens Opacity		58 (56.86%)	36 (55.38%)	22 (59.46%)	*χ*^2^ = 0.16	0.690
History of overexposure to radiation		2 (1.96%)	1 (1.54%)	1 (2.70%)		1.000^Δ^

^Δ^Fisher’s exact test; SD, Standard Deviation; Q1–Q3, the 1st quartile to the 3rd quartile; mSv, millisievert.

### Hematological differences

Males showed higher white blood cell (WBC) counts (6.09 ± 1.38 vs. 5.20 ± 1.25 × 10^9^/L, *p* = 0.002), higher red blood cell (RBC) counts (5.27 ± 0.27 vs. 4.47 ± 0.36 × 10^12^/L, *p* < 0.001) and hemoglobin concentrations (160.54 ± 7.58 vs. 133.49 ± 8.77 g/L, *p* < 0.001). However, No significant differences between males and females were observed in platelet counts or leukocyte differentials (Table 2).

**Table 2:** Results of laboratory test.

Parameter		All (*n* = 102)	Male	Female	Statistics	*p*-value
White Blood Cells Count (10^9^/L)	Mean (SD)	5.77 (1.39)	6.09 (1.38)	5.20 (1.25)	*t* = 3.21	0.002
Neutrophils (%)	Mean (SD)	58.58 (7.26)	58.39 (6.96)	58.90 (7.84)	*t* = −0.34	0.735
Lymphocytes (%)	Mean (SD)	33.13 (6.65)	33.15 (6.48)	33.08 (7.04)	*t* = 0.05	0.960
Monocytes (%)	Mean (SD)	6.12 (1.20)	6.25 (1.20)	5.89 (1.17)	*t* = 0.14	0.142
Eosinophils (%)	Mean (SD)	1.83 (1.19)	1.85 (1.25)	1.78 (1.09)	*z* = 0.15	0.751
	Median (Q1-Q3)	1.50 (0.90–2.48)	1.50 (0.90–2.50)	1.50 (0.80–2.40)		
Basophils (%)	Mean (SD)	0.31 (0.17)	0.30 (0.17)	0.34 (0.19)	*z* = −0.85	0.395
	Median (Q1-Q3)	0.30 (0.20–0.40)	0.30 (0.20–0.40)	0.30 (0.20–0.40)		
Red Blood Cells Count (10^12^/L)	Mean (SD)	4.98 (0.49)	5.27 (0.27)	4.47 (0.36)	*t* = 12.78	< 0.001
Hemoglobin (gram/L)	Mean (SD)	150.73 (15.32)	160.54 (7.58)	133.49 (8.77)	*t* = 16.36	< 0.001
Platelets Count (10^9^/L)	Mean (SD)	215.23 (40.84)	210.29 (41.72)	223.89 (38.26)	*t* = −1.63	0.106
	Median (Q1-Q3)	211 (184.25–243.75)	211 (178–239)	217 (197–244)		

SD, Standard Deviation; Q1-Q3, the 1st quartile to the 3rd quartile.

### Micronucleus abnormalities

The overall MN abnormality rate was 7.6 per 100 person-years and was markedly higher in female workers than in males (11.0 vs. 5.6; *z* = 2.65, *p* = 0.008). The median MN frequency was 0.004 for both sexes, However, females exhibited a wider interquartile range of MN frequency values (IQR 0.003–0.006 vs. 0.003–0.004; *z* = −3.86, *p* < 0.001).

The median cumulative effective dose during the study period was 0.44 mSv (IQR 0.11–0.69). Females received a significantly higher cumulative effective dose than males (0.57 vs. 0.36 mSv; *z* = −3.92, *p* < 0.001) (Table 3).

**Table 3:** Rate of micronucleus abnormalities and the exposure dose during the study period.

Metric	All (*n* = 102)	Male (*n* = 65)	Female (*n* = 37)	Statistic	*p*-value
Abnormality rate (per 100 person-years)	7.6	5.6	11.0	*z* = 2.65	0.008
MN/1000BN cells (per person per examination)					
Mean (SD)	4.09 (2.18)	3.88 (1.93)	4.46 (2.53)		
Median (Q1-Q3)	4 (3–5)	4 (3–4)	4 (3–6)	*z* = −3.86	< 0.001
Min-max	0–10	0–10	0–10		
Cumulative effective radiation dose during the study period (mSv) (per person per examination)					
Mean (SD)	0.70 (1.65)	0.69 (1.90)	0.70 (1.07)		
Median (Q1-Q3)	0.44 (0.11–0.69)	0.36 (0.08–0.64)	0.57 (0.33–0.80)	*z* = −3.92	< 0.001
Min ~ max	0.00–20.10	0.00–20.10	0.00–10.32		

BN, binucleated; MN, micronucleus; SD, standard deviation.

Based on these differences, we further examined potential determinants of MN abnormalities using Poisson regression analysis.

### Risk factors for micronucleus abnormalities

Control of Confounding Factors: We used a multivariate Poisson regression model and adjusted for age, years of employment in radiation-related work before the first examination, occupational categories (e.g., technician, nurse, physician), and cumulative radiation dose during the study period. Lifestyle factors, including smoking and alcohol consumption, were not included in the final multivariate model because they did not show a significant association with MN abnormalities in univariate analysis (*p* > 0.10). Furthermore, data on detailed medication use and quantitative measures of radiation protection compliance, such as dosimeter readings and protective equipment usage rates, were not available for analysis in this study.

We performed multivariate d Poisson regression analysis to identify independent predictors of MN abnormalities. The univariate analysis identified female sex (Coef. = 0.664, 95% CI: 0.207–1.120, *p* = 0.004) and hypothyroidism (Coef. = 2.064, 95% CI: 0.091–4.037, *p* = 0.040) to be significant positive predictors (Table 4). Subsequent multivariate analysis was conducted to control for potential confounders. In the multivariate model that adjusted for potential confounders, only female sex remained a significant and independent predictor of MN abnormalities. The adjusted coefficient was 0.636 (95% CI: 0.176–1.096, *p* = 0.007).

**Table 4:** Poisson regression for the risk of abnormal micronucleus.

Variable	Univariate		Multivariate	
	Coef. (95%CI)	*p*-value	Coef. (95%CI)	*p*-value
Year of examination	0.027 (−0.003, 0.057)	0.073	0.001 (−0.051, 0.053)	0.969
Sex (female)	0.664 (0.207 ~ 1.120)	0.004	0.636 (0.176, 1.096)	0.007
Educational level (master and above vs. bachelor vs. associate)	0.077 (−0.285, 0.440)	0.676		
Marriage (married)	0.978 (−0.029, 1.986)	0.057	0.798 (−0.271, 1.867)	0.143
Job rank (senior vs. intermediate vs. junior)	0.267 (−0.006, 0.540)	0.055	0.177 (−0.269, 0.622)	0.437
Employee classification (licensed physicians as ref.)				
Nurses	0.206 (−0.414, 0.826)	0.515		
Technologists	−0.310 (−0.861, 0.240)	0.269		
Years of radiation exposure before the initial examination	−0.004 (−0.038, 0.031)	0.841		
Smoke	−0.081 (−0.420, 0.259)	0.642		
Drink	−0.316 (−0.829, 0.197)	0.228		
Hashimoto’s thyroiditis	1.369 (−0.604, 3.342)	0.174		
Hypothyroidism	2.064 (0.091, 4.037)	0.040	1.393 (−0.625, 3.411)	0.176
Lens opacity	0.095 (−0.363, 0.553)	0.685		
Classification of radiographic equipment (Class III)	−0.124 (−0.581, 0.333)	0.595		
Cumulative radiation dose during the relevant examination	−0.766 (−1.921, 0.389)	0.194	−0.804 (−1.960, 0.352)	0.173
History of overexposure to radiation	0.318 (−0.690, 1.325)	0.537		

Coef., Coefficient; CI, confidential interval; mSv, millisievert.

However, several factors were not significantly associated with MN risk in the multivariate model. These included lifestyle factors such as smoking and alcohol consumption, occupational metrics like exposure duration and professional rank, and clinical factors including lens opacity (cataract). Notably, cumulative radiation dose was not a significant predictor either (Coef. = − 0.804, *p* = 0.173), and the negative coefficient suggests no biologically meaningful association in this context.

## Discussion

### Principal findings and interpretation

Our study identified a strong association between female sex and an elevated risk of MN abnormalities (≥6‰), independent of radiation dose, among medical radiation workers (22). This finding indicates that intrinsic biological susceptibility may play a more prominent role than small variations in physical dose in determining cytogenetic damage within the chronic low-dose occupational exposure range (23, 24). After establishing this central association, we examined lifestyle behaviors and cumulative radiation dose to contextualize the observed effect more fully.

### Contextualizing non-significant associations

The robustness of the sex-based disparity in MN abnormalities is underscored by the absence of significant associations with other measured variables, thereby weakening support for alternative explanations.

First, lifestyle factors such as smoking and alcohol consumption were reported only among male staff who exhibited lower MN rates. These factors were not significant predictors in the univariate analysis (Table 4) and were therefore not included as covariates in the final model (25–27). This suggests that, in our cohort, the genotoxic stress associated with chronic low-dose radiation outweighs the modest contributions of these behaviors (28).

In line with previous studies of chronic low-dose exposure (5), cumulative radiation dose was not independently associated with MN abnormalities in the multivariate model. This finding is particularly informative when combined with the observation that female workers received significantly higher cumulative doses than males, 0.57 vs. 0.36 mSv, *p* < 0.001. Despite these higher cumulative doses, females exhibited a markedly higher MN abnormality rate. The radiation dose was not an independent predictor in our model. Despite sexual dimorphism in exposure, this strongly suggests that the pronounced cytogenetic damage in females is not primarily driven by these small physical dose differences. Instead, it reinforces our central hypothesis that intrinsic biological susceptibility in females is a stronger risk factor for MN abnormalities than minimal variations in physical dose within this low-dose range. Rather than indicating a null dose-effect relationship, the absence of a measurable association likely reflects the fact that at very low exposure levels, inter-individual susceptibility becomes the most sensitive determinant of biological response. The MN assay is designed to capture this type of variation (2). Thus, the elevated MN rates among female workers are more plausibly attributable to intrinsic biological differences than to minor variations in exposure.

Furthermore, while discussing potential confounders, it is pertinent to address the observed sex-based differences in routine hematological parameters (Table 2). Males exhibited significantly higher counts of white blood cells (WBC), red blood cells (RBC), and hemoglobin (Hb) concentrations. These findings are consistent with established physiological norms and are likely influenced by the exclusive prevalence of smoking and alcohol consumption among male participants in our cohort. It is well-documented that smoking can elevate WBC, RBC, Hb, and mean corpuscular volume (MCV) (29, 30), while chronic alcohol consumption often leads to increased MCV and can decrease RBC and WBC counts (31, 32). Crucially, these hematological parameters reflect quantitative changes in blood cell populations and are distinct from the qualitative measure of chromosomal damage assessed by the MN assay. Our regression analysis confirmed that smoking and alcohol consumption were not significant direct predictors of MN abnormalities (Table 4). Therefore, we interpret the hematological differences as primarily reflecting physiological dimorphism and lifestyle factors, while the observed sex disparity in MN frequency is a separate phenomenon, more directly indicative of a differential cytogenetic response to chronic radiation exposure.

This compelling dose-independent disparity strongly points to underlying biological mechanisms as the primary explanation. We will explore the plausibility of these mechanisms in the following section.

### Cautions in interpreting the sex-based association: the role of confounding

Although the multivariate model identified female sex as a significant predictor of MN abnormalities, this association must be interpreted with caution due to substantial confounding.

First, we observe perfect collinearity between female sex and the absence of smoking and alcohol consumption in our cohort. Although these lifestyle factors were not significant predictors in univariate analysis, their complete separation by sex means that the variable “female” in our model effectively serves as a perfect proxy for “non-smoker” and “non-drinker,” thereby confounding interpretation. This multicollinearity challenges any definitive claim that the observed effect is solely attributable to biological sex, as it is statistically inseparable from these correlated behavioral patterns.

Second, occupational distribution differed sharply between sexes: most males were licensed physicians, while most females were nurses. Although we adjusted for broad occupational categories, it is unlikely that this fully captures differences in specific tasks, proximity to radiation sources, compliance with protective equipment, and exposure of unprotected body parts to scattered radiation (e.g., during patient care and positioning). Consequently, “female sex” may also serve as a proxy for a distinct set of occupational exposure patterns, which are not fully captured by personal dosimeter readings worn at the chest.

Thus, our findings likely reflect a composite effect of biological susceptibility, behavioral factors, and unmeasured occupational characteristics. The present study design does not allow these elements to be fully disentangled.

### Biological plausibility: elucidating the mechanisms of sex-based radiosensitivity

Despite the confounding issues outlined above, intrinsic biological differences may plausibly contribute to the observed disparity. We therefore present a hypothetical framework of potential mechanisms to be tested in future studies that better control non-biological factors. Given the dose-independent and lifestyle-independent association we observed, the strong link between female sex and MN abnormalities calls for a discussion of its biological plausibility based on existing literature. These mechanisms arise from intrinsic factors such as divergent DNA damage response capacities, genetic and epigenetic asymmetry, and oxidative stress management, and warrant further investigation (33–35).

### Hormonal regulation of DNA damage response

Estrogen signaling is hypothesized to exert a broad influence on genomic integrity by regulating key proteins involved in the repair of DNA double-strand breaks (DSBs), the most critical lesions induced by radiation. Core components of the homologous recombination pathway, such as BRCA1 and RAD51 are known targets of estrogen regulation (8, 36). Additionally, the base excision repair scaffold protein XRCC1 is also regulated by estrogen (9). In pre-menopausal women, this hormonal milieu may fine-tune the efficiency and fidelity of DSB repair. It is hypothesized that estrogen-mediated regulation could, under certain conditions, predispose female cells to slower or less accurate repair, thereby increasing the likelihood that misrepaired DSBs manifest as micronuclei (9, 37).

Supporting this hypothesis, recent experimental evidence shows that 17β-estradiol treatment modulates the transcription of DNA repair genes (38, 39), substantiating this link. These genes include XRCC5 and XRCC6, which are part of the non-homologous end joining pathway (38, 40). Furthermore, rapid non-genomic estrogen signaling modulates the phosphorylation and recruitment of DNA damage sensors such as ATM and *γ*-H2AX, potentially altering the early detection of radiation-induced DSBs (41). This complex hormonal influence may create a sexually dimorphic cellular environment (42), meaning a cellular environment that differs between sexes. In this environment, the coordination and kinetics of the DNA damage response differ, resulting in a greater load of persistent DNA damage and MN formation in females exposed to the same physical radiation dose (43, 44).

### X-chromosome-linked genetic and epigenetic asymmetry

The basic genetic differences between sexes may also contribute to this phenomenon. The X chromosome contains many genes involved in DNA repair and antioxidant defense. Notably, a significant proportion of these genes escape X-chromosome inactivation (45). This biallelic expression could change the balance of multi-protein DNA repair complexes, potentially creating a unique transcriptional profile for the DNA damage response in females (46).

For instance, the gene ATRX often escapes inactivation, and it is involved in chromatin remodeling and telomere maintenance. The biallelic expression of ATRX in females leads to significant differences in chromatin dynamics after irradiation (47). These differences, in turn affects the accessibility of DNA lesions to repair machinery.

### Possible divergence in management of radiation-induced oxidative stress

Well-documented sex differences in redox homeostasis (48) suggest that females may experience a distinct oxidative stress burden after exposure. While estrogen has antioxidant effects, it is also metabolized into catecholestrogens. These metabolites generate semiquinones and quinones, which lead to increased ROS production and oxidative DNA damage – a process called “estrogen genotoxicity” (49, 50). This pro-oxidant potential, combined with reported sexual dimorphism in antioxidant enzymes, could create a scenario in which the initial ROS burst from low-dose radiation triggers a more sustained damaging cycle in females, potentially fuelling micronuclei formation (51, 52).

### An integrated mechanistic perspective

In summary, we propose an integrative model: this model suggests that hormonal regulation, X-chromosome effects, and oxidative stress management work together to establish a sex-specific baseline for genomic integrity (23, 53, 54). When challenged by chronic radiation, this inherent susceptibility may be exacerbated, leading to the observed disparity. The model highlights specific, testable molecular pathways for future experimental validation, especially in populations occupationally exposed to radiation.

### Limitations

Our findings and the proposed biological mechanisms should be interpreted with caution. Several study limitations need to be taken into account. A primary limitation is that it lacks a matched control group of hospital workers who were not exposed. Since this study is retrospective and relies on existing occupational health records, it was not feasible to include a prospective unexposed control group. Without such a control group, accurately determining the baseline cytogenetic damage in the local population is difficult. Furthermore, identifying the exact proportion of MN abnormalities caused solely by occupational radiation exposure, as opposed to environmental, lifestyle, or genetic factors, remains challenging. We used a standardized MN frequency cut-off (≥ 6‰) based on national norms and prior studies to define abnormality. However, an internal control group would have offered a more direct and context-specific comparison.

The most significant limitation is the final sample size. It fell short of our *a priori* target and resulted in a notably underpowered female subgroup (*n* = 37). Although a post-hoc analysis indicated acceptable power (0.78) for detecting the primary effect of sex, this power was still below the conventional 0.80 threshold. Nevertheless, while we observed a statistically significant association, the robustness of our point estimate for female sex (Coef. = 0.636) requires verification in a larger cohort. The limited statistical power of the study also affects the interpretation of non-significant findings. Notably, the absence of a significant association between cumulative radiation dose and MN abnormalities should be interpreted with extreme caution. It could either reflect a true lack of association within this low-dose range or be a false negative due to limited power to detect weaker effects. Therefore, the central finding of sex-based differences, though robust in our model, should be seen as suggesting a plausible hypothesis rather than providing a definitive estimate of the effect.

Third, the most critical issue for interpreting our primary finding is the profound confounding between sex and sociodemographic/occupational variables. In our cohort, none of the females smoked or consumed alcohol, and they were strongly associated with specific occupational roles, such as a predominance of nurses. This severe multicollinearity means that the variable ‘female sex’ in our model likely acts as a proxy. It represents a complex combination of biological, behavioral, and occupational factors that we could not fully analyze. Although we adjusted for broad occupational categories and found lifestyle factors to be non-significant in univariate analysis, extensive residual confounding remains a major limitation. This issue strongly challenges any definitive causal interpretation attributing the observed association solely to biological sex, rather than to these closely related and sometimes unmeasured factors.

Fourth, the study’s retrospective design precluded repeated biomonitoring. The MN frequency was determined from a single blood sample per individual, which constitutes a major methodological constraint. A single time-point measurement may vary due to intra-individual biological changes over time (e.g., short-term changes in health, immune activity, or stress). This variability can cause non-differential misclassification. As a result, observed associations may be biased toward the null, potentially underestimating true effect sizes, for example, the effect size associated with female sex. We attempted to minimize diurnal variation by conducting most examinations in the morning and adhered to a strict standardized protocol with blinded scoring. However, the inherent instability of a single measurement remains a fundamental limitation to the precision of our risk estimates. Therefore, future prospective studies should incorporate a repeated-measures design (e.g., a second MN assay 1–2 years after baseline). This approach would allow quantification of variability using the intra-class correlation coefficient (ICC) and enable calculation of mean values or application of longitudinal data analysis methods to provide a more reliable assessment of an individual’s baseline chromosomal damage and its long-term trajectory.

Fifth, our assessment of non-occupational confounders was limited. Although we accounted for major lifestyle factors such as smoking and alcohol consumption—both of which showed no significant association with MN outcomes in our cohort—we lacked data on other potential sources of genotoxic exposure. These sources include hobbies (e.g., involving solvents, paints, or woodworking), dietary patterns (such as consuming charred meats or antioxidant-rich foods), and detailed residential environmental factors. To address this gap, future studies should incorporate a detailed lifestyle and environmental exposure questionnaire that carefully documents hobbies, dietary patterns, use of personal care products, and residential history. The lack of this information may lead to unmeasured confounding, since these factors could contribute to baseline cytogenetic damage. However, because our cohort consisted of health professionals with likely similar socioeconomic status and health awareness, large-scale differences in such uncommon exposures between male and female workers are unlikely. Nevertheless, the influence of these factors cannot be entirely ruled out.

Finally, the proposed biological mechanisms remain speculative because no direct molecular data are available. Such data would include hormonal profiles; measures of DNA repair function, such as those obtained via *γ*-H2AX foci assays, oxidative stress biomarkers; and genetic polymorphisms. This study was designed to identify an epidemiological association rather than to validate the underlying molecular pathways.

### Public health implications and future directions

Despite these limitations, our study provides preliminary evidence. If validated, this evidence carries significant implications for occupational radiation protection.

Our findings carry immediate implications for occupational radiation protection standards and practices. The core principles of international frameworks—justification, optimization (also known as ALARA principle), and dose limitation—face challenges. This challenge arises due to evidence showing significant, dose-independent biological variation between sexes. We have identified being female as a major risk factor for cytogenetic damage. This finding necessitates a re-evaluation of the current “one-size-fits-all” approach. To address this, we propose the following concrete steps to integrate sex as a biological variable into safety protocols:

Refinement of the ALARA principle: Protection strategies for female workers should be intensified, especially for those with MN frequencies near the abnormality threshold. This could include prioritized access to advanced personal protective equipment, stricter adherence to shielding protocols, and adjusting job rotation schedules to minimize cumulative exposure time.

Biological monitoring should be integrated because physical dosimetry alone cannot reveal intrinsic biological susceptibility. Thus, occupational health policies should emphasize the importance of regular biological monitoring. For example, annual or biennial MN assays can complement physical dose measurements to detect health risks at an early stage.

Updated Training and Education: Radiation protection training programs must now include education on sex-based differences in radiosensitivity. This is essential to equip all healthcare workers and safety officers with the knowledge to implement personalized and effective protection strategies. Hence, formally incorporating these sex-specific considerations is a necessary step toward advancing health equity and safeguarding the well-being of all healthcare professionals.

### Future research directions

To convert our epidemiological findings into practical insights and to address remaining mechanistic questions, we propose the following research priorities.

#### Enhanced study design

Future investigations should use a prospective, multicenter approach that includes a matched internal control group of non-exposed healthcare workers (e.g., administrative staff or unexposed clinical personnel from the same hospital system). This is crucial for establishing a context-specific baseline of cytogenetic damage, quantifying radiation-attributable risk, and ensuring balanced occupational representation between sexes.

#### Comprehensive exposure assessment

Future studies should use more detailed lifestyle and environmental questionnaires. These tools should systematically document hobbies, dietary habits, medication use, and residential history to better control potential non-occupational sources of genotoxic stress.

#### Methodological innovations to address confounding

Future studies should adopt specific methods to separate sex effects from related confounders. Techniques such as propensity score matching should be used to create balanced cohorts of male and female workers with similar occupational profiles and lifestyle habits. Alternatively, larger studies should be planned in advance to recruit equal numbers of male and female workers across job categories, this approach will enable a clearer distinction between biological sex and occupational or behavioral effects.

#### Elucidation of underlying mechanisms

Research must include direct molecular measurements to test the biological mechanisms proposed in this study. These measurements should involve assays for sex hormone levels, DNA repair capacity (e.g., *γ*-H2AX foci assay), oxidative stress biomarkers, and genotyping for relevant genetic polymorphisms. Additionally, *in vitro* models should be employed to directly test the causal effects of sex hormones on radiation-induced cytogenetic damage.

It is now essential to include biological sex as a fundamental variable in the design and analysis of future occupational radiation studies. This inclusion is necessary to develop a precise and equitable understanding of radiation risk.

## Conclusion

In summary, this retrospective cohort study identified a significant relationship between female sex and an increased risk of micronuclei abnormalities. This correlation was observed specifically among healthcare workers exposed to chronic low-dose radiation. However, this observed relationship is confounded by strong correlations with occupational roles and lifestyle factors, precluding definitive conclusions that biological sex alone drives the effect. If validated in larger, prospective studies specifically designed to disentangle these effects, this finding would underscore the necessity of moving beyond a purely dose-focused model. Future risk assessment frameworks should consider the complex interplay of sex, occupation, and lifestyle factors to develop more personalized and equitable safety standards for the global medical radiation workforce.

## Data Availability

The raw data supporting the conclusions of this article will be made available by the authors, without undue reservation.
